# Ossification of the Posterior Longitudinal Ligament in the Cervical, Thoracic, and Lumbar Spine

**DOI:** 10.7759/cureus.14041

**Published:** 2021-03-22

**Authors:** Adriel Barrios-Anderson, Elaina J Wang, Rahul Sastry, Jared S Fridley

**Affiliations:** 1 Neurosurgery, The Warren Alpert Medical School of Brown University, Providence, USA; 2 Neurological Surgery, The Warren Alpert Medical School of Brown University, Providence, USA

**Keywords:** ossification of the posterior longitudinal ligament, myelopathy, spinal cord injury, ossification, spine

## Abstract

Ossification of the posterior longitudinal ligament (OPLL) is a relatively rare disorder characterized by elongation of the posterior longitudinal ligament followed by the progressive development of ectopic osseous tissue along the ligament. OPLL is most commonly reported in the cervical spine, with fewer reported cases of thoracic or lumbar OPLL. The incidence of OPLL is high in east Asian populations with a much lower incidence in the United States. In this case report and review, we present the case of a 44-year-old female who was admitted to the hospital with a one-year history of progressive bilateral lower extremity weakness. Her lower extremity weakness had worsened over months and precipitated a gait disturbance that left her wheelchair-bound at the time of presentation. Additional presenting symptoms included lower back pain, stool incontinence, neck pain, and upper extremity paresthesias. Computed tomography of the spine revealed multiple areas of osteophyte formation and OPLL in the cervical spine from C2-5, thoracic spine from T6-10, and in the lumbar and sacral spine from L1-S1. There were notable areas of accompanying neural foraminal stenosis and central canal stenosis with visible spinal cord compression present in various locations. The patient did not undergo surgical intervention given the significant risk of multilevel surgery, and her symptoms were managed with medication. OPLL, particularly when not considered in lower-risk populations, can be a significant cause for progressive debilitating neurological abnormality. We report a rare case of OPLL occurring throughout the cervical, thoracic, lumbar, and sacral spine.

## Introduction

Ossification of the posterior longitudinal ligament (OPLL) is a relatively rare condition of unknown etiology that is marked by the spontaneous development of ectopic osseous tissue along the posterior longitudinal ligament of the spinal cord [1,2]. The pathophysiology of OPLL is not completely understood, and while various genetic associations and biomarkers have been associated with the disease, a clear understanding of the disease remains elusive [3,4]. OPLL often affects the cervical spine, and progression of the disease can cause cervical myelopathy and significant morbidity due to spinal cord injury [1]. The presentation and natural progression of OPLL are varied but often include symptoms consistent with spinal radiculopathy and myelopathy at various spinal levels.

The surgical treatment of cervical OPLL is a subject of debate among experts and is largely approached on an individual basis based upon the symptoms or risk of severe spinal cord injury, risks of surgical intervention, and patient-specific considerations [5,6]. Treatment is further complicated by the increased risk of certain diseases highly associated with OPLL such as idiopathic skeletal hyperostosis and ankylosing spondylitis, and the relatively high incidence of concomitant thoracolumbar OPLL involvement [6,7]. The literature is markedly limited in discussing the appropriate surgical and medical management of complicated presentations of OPLL involving multiple sites along the spinal cord.

Here, we present the case of a 44-year-old woman with OPLL of the cervical, thoracic, and lumbar spine. We also provide a review of recent literature on the subject of OPLL with special consideration to management options and discussion when the pathology may or may not be appropriate for surgical intervention.

## Case presentation

A 44-year-old female presented to our hospital with a decline in lower extremity strength and ambulation ability over nine months. She also reported increased numbness of the lower extremities, worsening lower back pain, bowel incontinence, neck pain, and paresthesias, numbness, and weakness of the upper extremities. Medical history included severe diabetes mellitus complicated by peripheral neuropathy and a foot ulcer and chronic urinary incontinence treated with a bladder stimulator.

Neurologic examination revealed a motor and sensory deficit in the lower limbs with 3 out of 5 muscle strength and loss of sensation to light touch in the bilateral lower extremities up to the level of the upper thighs. The upper extremities showed 4 out of 5 strength bilaterally, with a notable claw hand deformity of the left hand. Sensation to light touch was diminished in the distal upper extremities.

**Figure 1:**
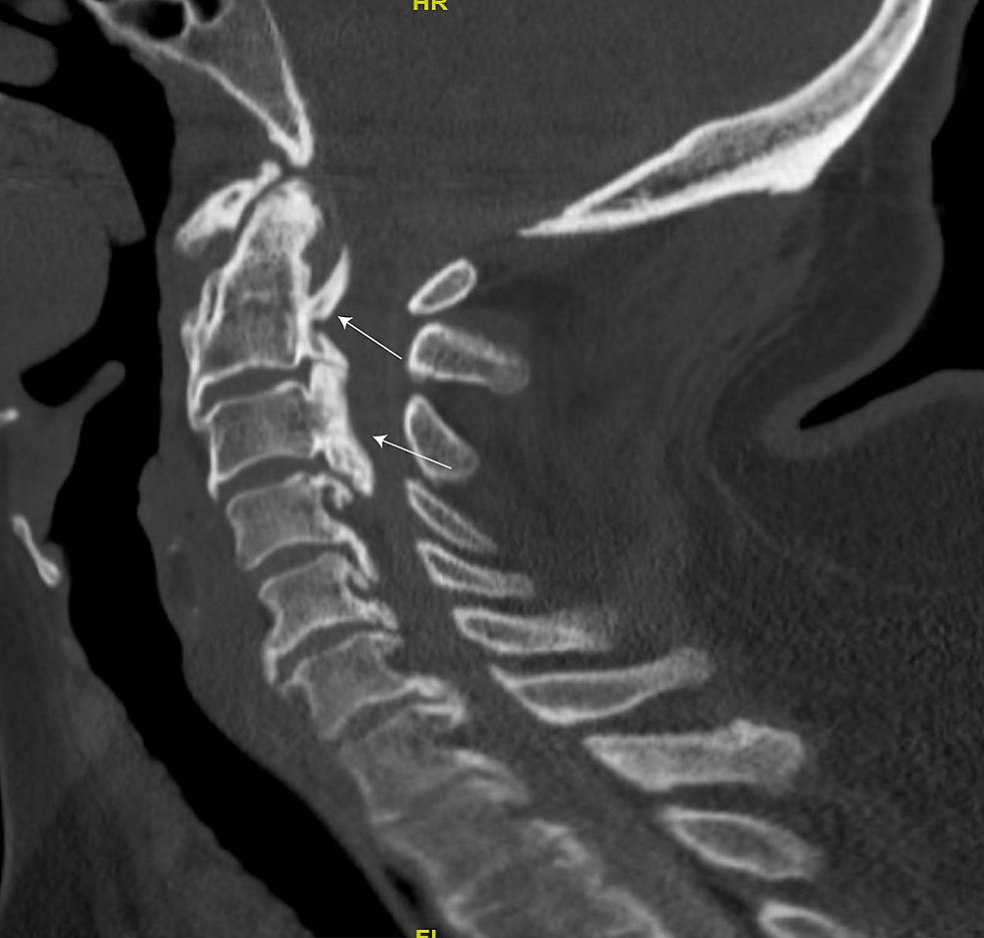
Sagittal CT myelogram demonstrating OPLL from C2-5 in the cervical spine. The arrows indicate few areas of ossification visible on the image slice. CT, computed tomography; OPLL, ossification of the posterior longitudinal ligament

**Figure 2:**
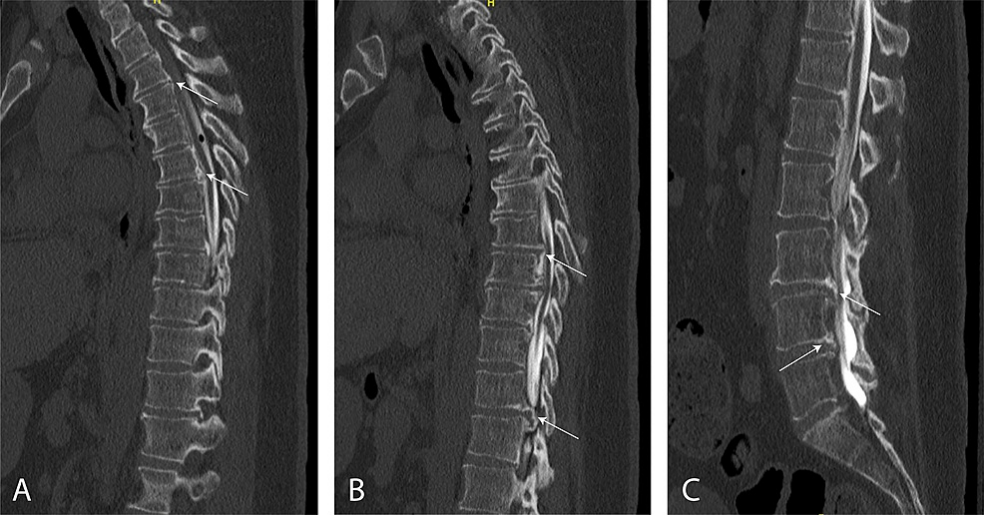
Sagittal CT myelogram demonstrating OPLL in the thoracic (A, B) and lumbosacral (C) spine from T2-5, T6-10, and L1-S1. The arrows indicate few areas of ossification visible on the image slice. CT, computed tomography; OPLL, ossification of the posterior longitudinal ligament

**Figure 3:**
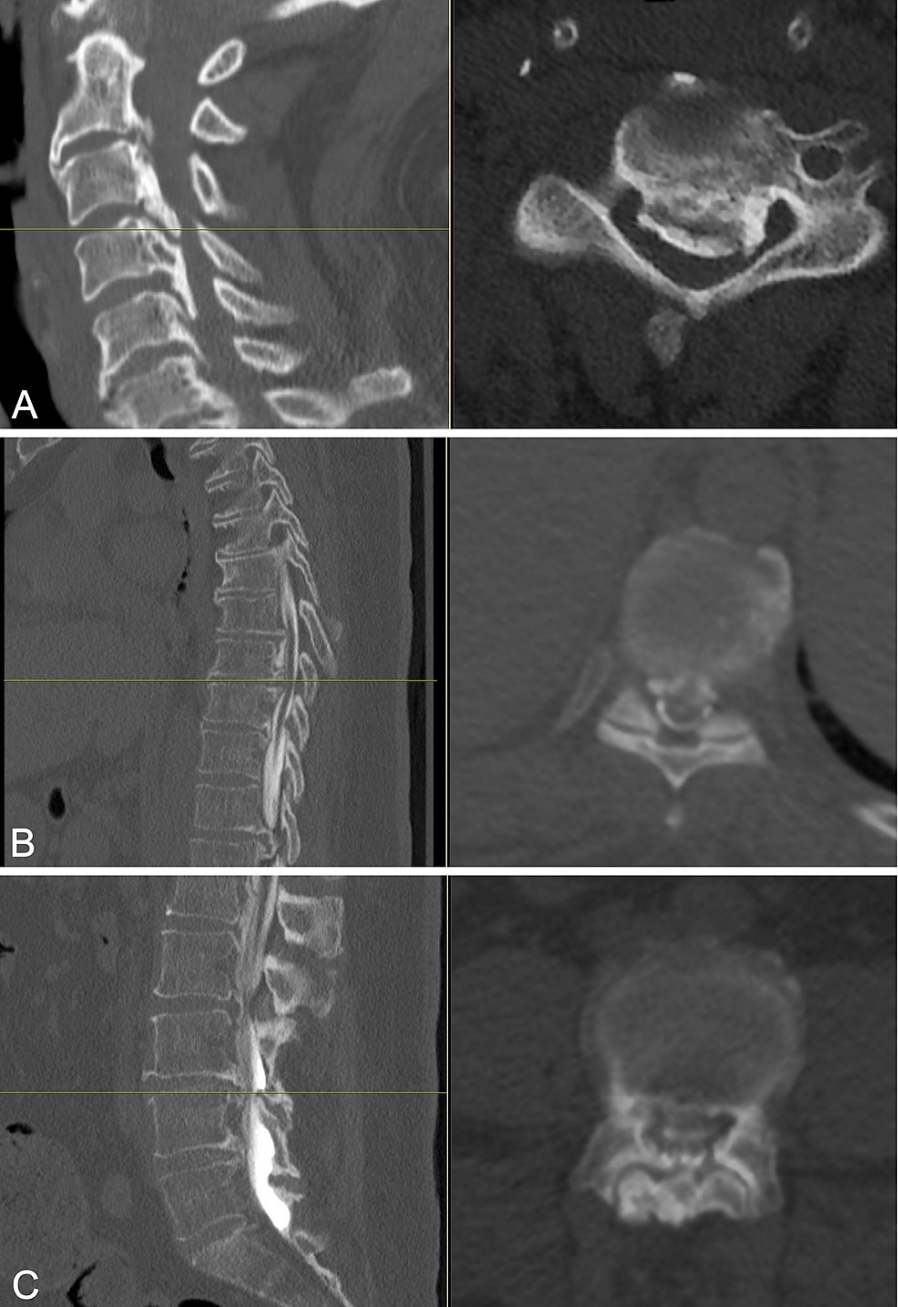
Sample axial CT myelogram images demonstrating degree of stenosis in the cervical (A), thoracic (B), and lumbar (C) spine. The horizontal yellow line in the left panel shows the location in the sagittal view of the corresponding axial slice shown in the adjacent right panel. The degree of stenosis is visible on the sample axial slices at C3-4 (A), T9-10 (B), and L3-4 (C). CT, computed tomography

Computed tomography (CT) of the cervical, thoracic, and lumbar spine demonstrated extensive OPLL throughout the length of the spinal cord (Figures 1, 2, 3). Specifically, prominent osteophytes causing severe central canal stenosis were identified at C3-5, T6-10, and L3-4. Severe bilateral neural foraminal stenosis was also present in both the cervical and lumbar spine. The patient was not offered surgical intervention given her multiple medical comorbidities coupled with the risks of the complex multi-stage procedure that would be needed to address her extensive OPLL. Further, it was determined that given the progression of her disease at presentation, surgical intervention would offer only minimal functional improvement if successful. The patient was offered a regimen of duloxetine, gabapentin, tramadol, and acetaminophen for chronic back pain management and opted for conservative management.

## Discussion

The pathophysiology of cervical OPLL is not well established. Several studies have demonstrated a significant genetic component [3]. OPLL has been linked to inflammatory markers such as erythrocyte sedimentation rate and high-sensitivity C-reactive protein, although it remains controversial whether inflammation is a cause or effect of OPLL [4]. Cervical OPLL is one of the main causes of cervical myelopathy, the most common form of myelopathy in adults [8]. It is considered to have a higher prevalence in Asian countries compared to the general population, with a prevalence of 2-4% in Japan compared to 0.01-2% in non-Asian populations [8]. CT findings analyzed from a 1,500 patient sample in Japan demonstrated a cervical OPLL prevalence of 8.3% and 3.4% in men and women, respectively, compared to a thoracolumbar OPLL prevalence of 1.4% and 2.0% in men and women, respectively [9].

Notably, in both men and women, the number of cervical levels affected in patients with cervical OPLL is correlated with an increased risk of concomitant thoracic or lumbar OPLL [10]. Kawaguchi et al. demonstrated that in a group of 178 patients with cervical OPLL, 25.8% had concomitant cervical and thoracic OPLL, 18.5% had cervical and thoracolumbar OPLL, and 9% had cervical and lumbar OPLL [11,12]. Further analysis in the same study revealed that while the prevalence of OPLL was higher in males, the prevalence of severe OPLL involving multiple levels was higher in females [11,12]. Here, we demonstrate a relatively rare case of extensive OPLL in the cervical, thoracic, lumbar, and sacral spine of a female patient.

Cervical OPLL typically presents with symptoms of cervical radiculopathy and myelopathy [1]. Given that thoracic OPLL is less prevalent than cervical OPLL, there are fewer cross-sectional studies regarding the clinical manifestations of thoracic OPLL. A 2018 case report described a patient with thoracic OPLL who presented with bilateral lower leg weakness, exaggerated lower deep tendon reflexes, upgoing Babinski sign, and progressive unsteadiness [13], which is consistent with symptoms of thoracic myelopathy.

Surgical intervention has demonstrated considerable efficacy in improving clinical outcomes for patients with myelopathy as a result of cervical or thoracolumbar OPLL [6,14]. The degree of myelopathy, determined by clinical symptom severity, is associated with the potential benefit of surgery with severe cases showing benefit from surgery, while medical management offers the same benefit as surgery for those with minimal symptoms [6,15]. For those treated conservatively, however, the risk for significant spinal cord injury causing significant disability remains markedly elevated [6,16]. Reported surgical applications for OPLL aim to decompress the spinal cord and subsequently stabilize the spinal column via different approaches including anterior cervical discectomy and fusion, posterior laminectomy and fusion, posterior laminoplasty, and circumferential approaches such as posterior circumferential decompression [6,7,14,17]. Different approaches have been reported with variable success in OPLL at distinct spinal levels; however, none is without complications, and the optimal approach for OPLL in the cervical, thoracic, or lumbar spine remains controversial [6,14]. To the best of our knowledge, this is the first case report of a multi-level OPLL medical approach explained in English literature. No reported literature, to our knowledge, has established optimal surgical strategies for extensive multi-level OPLL as presented in this case.

## Conclusions

This case report describes a rare occurrence of OPLL at various spinal levels involving the cervical, thoracic, lumbar, and sacral spine. The radiographic and clinical severity of the case along with existing comorbidities led to the ultimate decision for conservative, medical management of this patient’s progressive disease. Existing literature on OPLL suggests that the etiology, pathophysiology, and best management of this rare, progressive disease remains controversial. Surgeons must continue to evaluate clinical severity and determine the best surgical approaches when appropriate on a case-by-case basis for patients with extensive, multi-level OPLL.
